# MIEF2 reprograms lipid metabolism to drive progression of ovarian cancer through ROS/AKT/mTOR signaling pathway

**DOI:** 10.1038/s41419-020-03336-6

**Published:** 2021-01-05

**Authors:** Shuhua Zhao, Lu Cheng, Yuan Shi, Jia Li, Qinghui Yun, Hong Yang

**Affiliations:** 1 Department of Gynaecology and Obstetrics, Xijing Hospital, Fourth Military Medical University, Xi’an, China; 2 Department of medical equipment, Xijing Hospital, Fourth Military Medical University, Xi’an, China

**Keywords:** Ovarian cancer, Ovarian cancer

## Abstract

MIEF2 (mitochondrial elongation factor 2) is one of the key regulators of mitochondrial fission. Bioinformatics analysis indicated that high expression of MIEF2 predicted a poor prognosis in ovarian cancer patients. However, the relationship between MIEF2 and aberrant lipid metabolism in OC remains elusive. In this study, we demonstrated that MIEF2 significantly promoted lipid synthesis, while has no significant effect on fatty acid uptake and oxidation in OC cells. MIEF2 enhanced de novo fatty acid synthesis through up-regulating the expression of sterol regulatory element binding protein 1 (SREBP1) and its transcriptional target lipogenic genes ACC1, FASN and SCD1. Meanwhile, MIEF2-promoted cholesterol biosynthesis through up-regulating the expression of sterol regulatory element binding protein 2 (SREBP2) and its transcriptional target cholesterol biosynthesis genes HMGCS1 and HMGCR. Mechanistically, increased mitochondrial reactive oxygen species (ROS) production and subsequently activation of AKT/mTOR signaling pathway was found to be involved in the up-regulation of SREBP1 and SREBP2 in OC cells. Moreover, cell growth and metastasis assays indicated that MIEF2-regulated fatty acid synthesis and cholesterol biosynthesis played a critical role in the progression of OC. Taken together, our findings indicate that MIEF2 is a critical regulator of lipid synthesis in OC, which provides a strong line of evidence for this molecule to serve as a drug target in the treatment of this malignancy.

## Introduction

Reprogrammed fatty acid metabolism mainly characterized by increased de novo lipgenesis has been increasingly established as a hallmark of cancer. Elevated lipgenesis, including de novo fatty acid synthesis^1,2^ and cholesterol biosynthesis^3,4^, provides cancer cells with building blocks, signaling molecules and post-translational modifications to promote tumor growth and metastasis. Besides, increased de novo lipogenesis in cancer cells also makes them more independent from externally provided lipids^5^. Additionally, fatty acid also serves as an important energy source during energy stress through mitochondria-mediated β-oxidation^6^.

Increased fatty acid synthesis and cholesterol biosynthesis, as well as fatty acid oxidation are supported by enhanced expression of the enzymes belonging to these pathways, which are transcriptionally regulated by the sterol regulatory element-binding protein 1 (SREBP1), sterol regulatory element-binding protein 2 (SREBP2) and peroxisome proliferator-activated receptors (PPARs), respectively^7,8^. To date, increased expressions of many enzymes involved in fatty acid synthesis and cholesterol biosynthesis, such as acetyl-CoA carboxylase (ACC)^9^, fatty acid synthase (FASN)^1^ and stearoyl-CoA desaturase1 (SCD1)^10^, 3-hydroxy-3-methyl-glutarylcoenzyme A reductase (HMGCR)^11^, 3-Hydroxy-3-methylglutaryl coenzyme A (CoA) synthase (HMGCS)^12^, have been observed in many different types of cancer. As major transcription factors that control the expression of enzymes involved in fatty acid and cholesterol biosynthesis, increased expressions and transcriptional activities of SREBP1 and SREBP2, which contributed to tumor progression, have also been demonstrated in several cancer types^13–15^. Cumulative evidence indicates that SREBPs are principally activated by the oncogenic signaling pathway Akt/mTOR (mammalian target of rapamycin complex 1), promoting the nuclear accumulation of SREBPs and thus driving lipid synthesis during tumor progression^16–19^. During recent years, there has been a revival of enthusiasm amongst investigators to investigate how lipid metabolism is reprogrammed in cancer cells. However, its underlying mechanisms remains not completely understood.

Mitochondria are crucial organelles involved in cellular metabolism regulation, the morphology of which is dynamically remodeled fission and fusion events^20–22^. During recent years, dysregulation of mitochondrial fission and fusion dynamics has been revealed in various types of human cancers, which contributed to the progression of cancer^23–25^. MIEF2 (mitochondrial elongation factor 2) is one of the key regulators of mitochondrial fission^26^. Using the online Kaplan–Meier plotter (http://kmplot.com/analysis/), we found that MIEF2 high expression predicts a significant poor prognosis (HR = 1.36, 95% CI: 1.10–1.7, *P* = 0.0036) in patients with OC (Supplementary Fig. S1A), suggesting a potential oncogenic role of MIEF2 in the progression of ovarian cancer (OC). However, the role of MIEF2 in lipid metabolism reprogramming of cancer cells is still largely unclear.

In the present study, we systematically explored the role and underling molecular mechanisms of MIEF2 in the reprogramming of lipid metabolism in OC cells.

## Results

### MIEF2 markedly elevated the lipid content in ovarian cancer cells

**Fig. 1:**
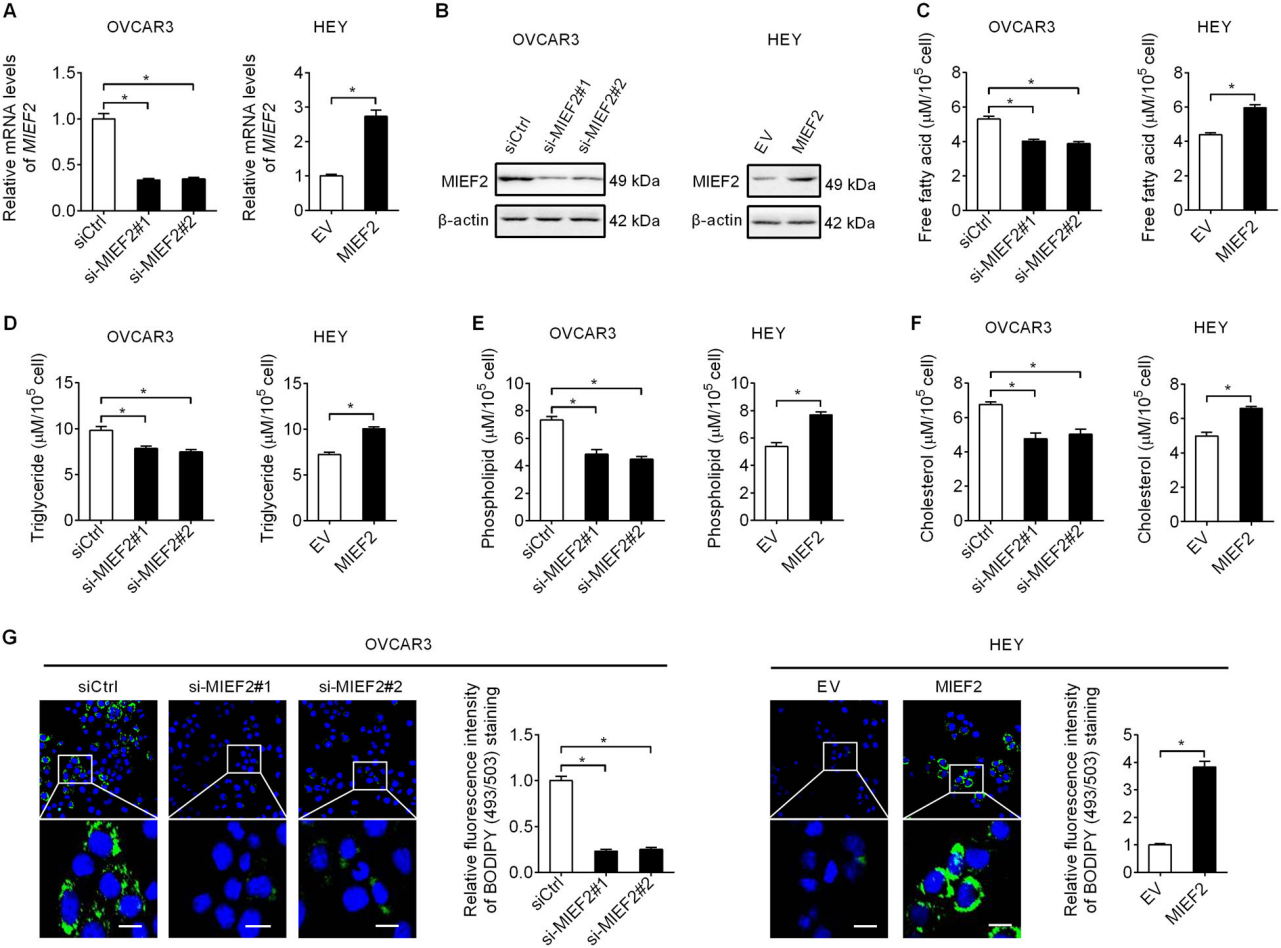
MIEF2 markedly elevated the lipid content in ovarian cancer cells. **a** Knockdown or over-expression of MIEF2 in OVCAR3 and HEY cells were verified by quantitative real-time PCR analysis. (siMIEF2–1 and siMIEF2–2, siRNAs against MIEF2; siCtrl, control siRNA; MIEF2, expression vector encoding MIEF2; EV, empty vector; *N* = 3). **b** Knockdown or over-expression of MIEF2 in OVCAR3 and HEY cells were verified by western blot analysis. **c**–**f** Intracellular levels of free fatty acid (**c**), triglyceride (**d**), phospholipids (**e**) and cholesterol (**f**) were measured in OVCAR3 and HEY cells with MIEF2 knocked-down or over-expressed (*N* = 3). **g** Detection of neutral lipid content by staining with fluorescence dye BODIPY 493/503 in OVCAR3 and HEY cells with MIEF2 knocked-down or over-expressed. Scale bars, 20 μm. The relative average fluorescence intensity per cell was analyzed with image J (*N* = 20 cells per group).

Using the online Kaplan-Meier plotter^27^ analysis, we found that MIEF2 high expression predicted a significant poor prognosis (HR = 1.36, 95% CI: 1.10–1.7, *P* = 0.0036) in patients with OC (Supplementary Fig. S1A), suggesting a potential oncogenic role of MIEF2 in the progression of OC. To study the role of MIEF2 in the aberrant lipid metabolism of OC cells, we set out to measure the changes of lipid content in OVCAR3 cells with relative higher MIEF2 expression level and in HEY cells with relative low MIEF2 expression level (Supplementary Fig. S1B and S1C). The successful knockdown or overexpression of MIEF2 was evidenced by qRT-PCR and Western blot analysis (Fig. 1a, b and Supplementary Fig. S1D). We found that knockdown of MIEF2 in OVCAR3 cells significantly decreased levels of intracellular free fatty acid (Fig. 1c), triglyceride (Fig. 1d), phospholipids (Fig. 1e) and cholesterol (Fig. 1f), while overexpression of MIEF2 markedly increased the levels of those lipids in HEY cells (Fig. 1c–f). Consistently, intracellular neutral lipids staining with fluorescence lipophilic dye BODIPY 493/503 in OVCAR3 and HEY cells also indicated that MIEF2 significantly elevated the intracellular contents of neutral lipids in OC cells (Fig. 1g). Together, these data strongly demonstrate that MIEF2 plays a dominant role in regulating lipid metabolism in OC cells.

### MIEF2 increased the expression levels of lipogenic enzymes in OC cells

**Fig. 2:**
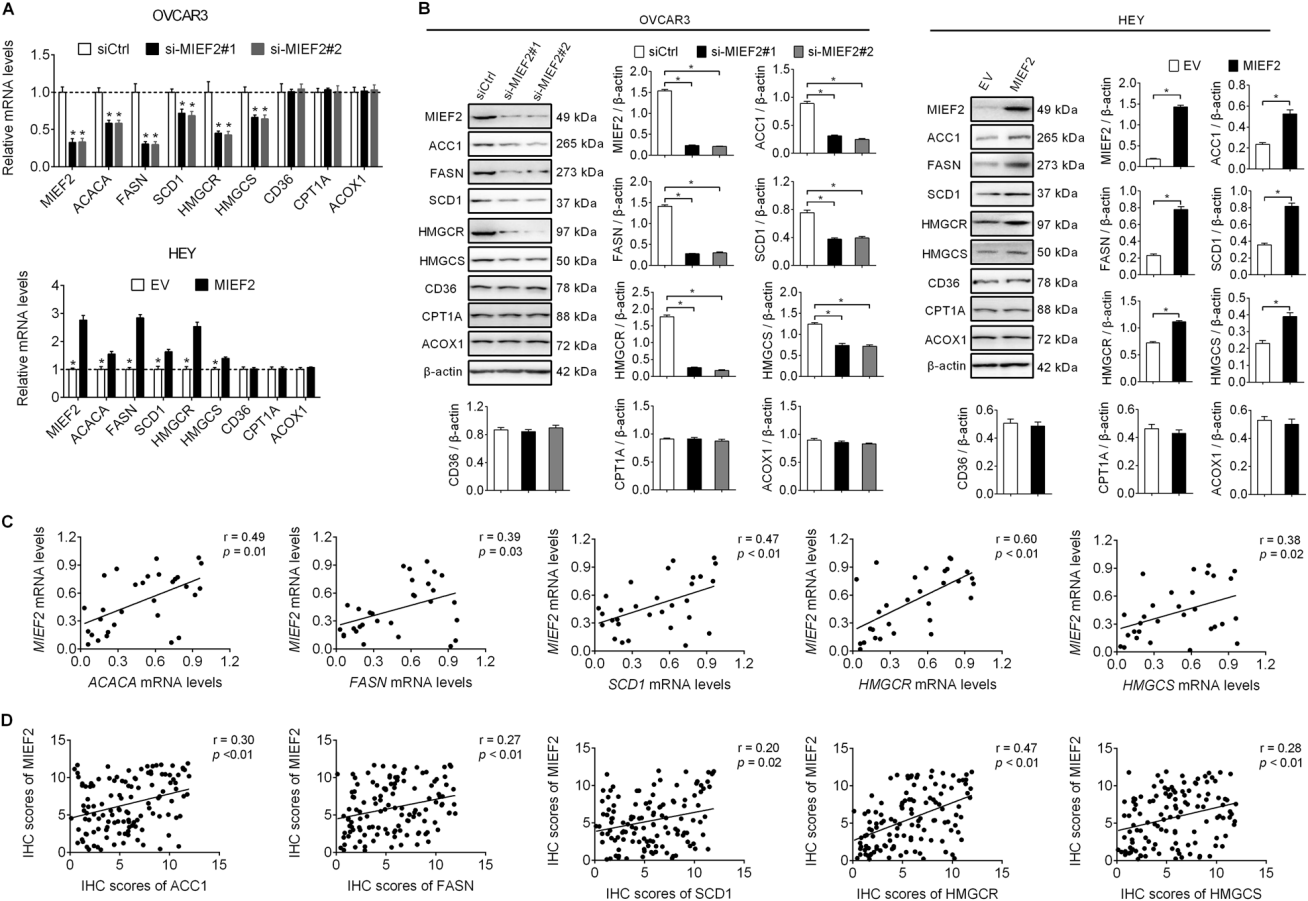
MIEF2 increased the expression levels of lipogenic enzymes in OC cells. **a**, **b** Quantitative RT-PCR and western blot analysis for mRNA and protein expression levels of *de novo* fatty acid synthesis enzymes of ACC1, FASN and SCD1, cholesterol biosynthesis enzymes of HMGCS1 and HMGCR, fatty acid uptake CD36 and fatty acid oxidation enzymes of CPT1A and ACOX1 in OVCAR3 and HEY cells with MIEF2 knocked-down or over-expressed (*N* = 3). **c** Spearman correlation analysis of the relationship between the mRNA expression levels of MIEF2 and lipogenic enzymes (*ACACA, FASN, SCD1, HMGCS1* and *HMGCR*) in tumor tissues from 30 OC patients. **d** Spearman correlation analysis of the relationship between the protein expression levels of MIEF2 and lipogenic enzymes (ACC1, FASN, SCD1, HMGCS1 and HMGCR) in tumor tissues from another 122 OC patients.

Increased cell lipid content could be caused by accelerated lipid biosynthesis, increased fatty acid uptake and decreased lipid catabolism. Therefore, the expression levels of key molecules involved in fatty acid synthesis (ACC1, FASN, SCD1), cholesterol biosynthesis (HMGCS1, HMGCR), fatty acid uptake (CD36) and fatty acid oxidation (CPT1A) in OVCAR3 and HEY cells were firstly determined when MIEF2 was knocked-down or over-expressed. As shown in Fig. 2a, b, MIEF2 knockdown markedly suppressed the expression levels of both fatty acid synthesis and cholesterol biosynthesis enzymes in OVCAR3 cells, while the levels of key factors involved in fatty acid uptake and oxidation were unchanged. Conversely, MIEF2 over-expression in HEY cells resulted in significantly increased expression levels of those lipogenic enzymes. These data indicate that MIEF2 increases *de novo* fatty acid synthesis and cholesterol biosynthesis, while has no effect on fatty acid uptake and oxidation, which was further supported by the assessment of the rates of fatty acid uptake and oxidation using ^3^H-labeled oleic acid as a tracer (Supplementary Fig. S2A and S2B). To provide further support, the expression levels of MIEF2 and lipogenic enzymes of ACC1, FASN, SCD1, HMGCS1 and HMGCR were determined by qRT-PCR in tumor tissue samples from 30 OC patients. Spearman rank correlation analysis indicated significantly positive correlations between the expression levels of MIEF2 and lipogenic enzymes of ACC1 (*r* = 0.49, *p* = 0.01), FASN (*r* = 0.39, *p* = 0.03), SCD1 (*r* = 0.47, *p* < 0.01), HMGCS1 (*r* = 0.60, *p* < 0.01) and HMGCR (*r* = 0.38, *p* < 0.02) (Fig. 2c). Similar results were also obtained from another 122 OC patients by immunohistochemistry staining assay (Supplementary Fig. S3), which further confirmed the significantly positive correlations between the expression levels of MIEF2 and lipogenic enzymes of ACC1 (*r* = 0.30, *p* < 0.01), FASN (*r* = 0.27, *p* < 0.01), SCD1 (*r* = 0.20, *p* = 0.02), HMGCS1 (*r* = 0.47, *p* < 0.01) and HMGCR (*r* = 0.28, *p* < 0.01) (Fig. 2d).

### MIEF2 increased the expression levels of fatty acid synthesis enzymes via up-regulating SREBP1

**Fig. 3:**
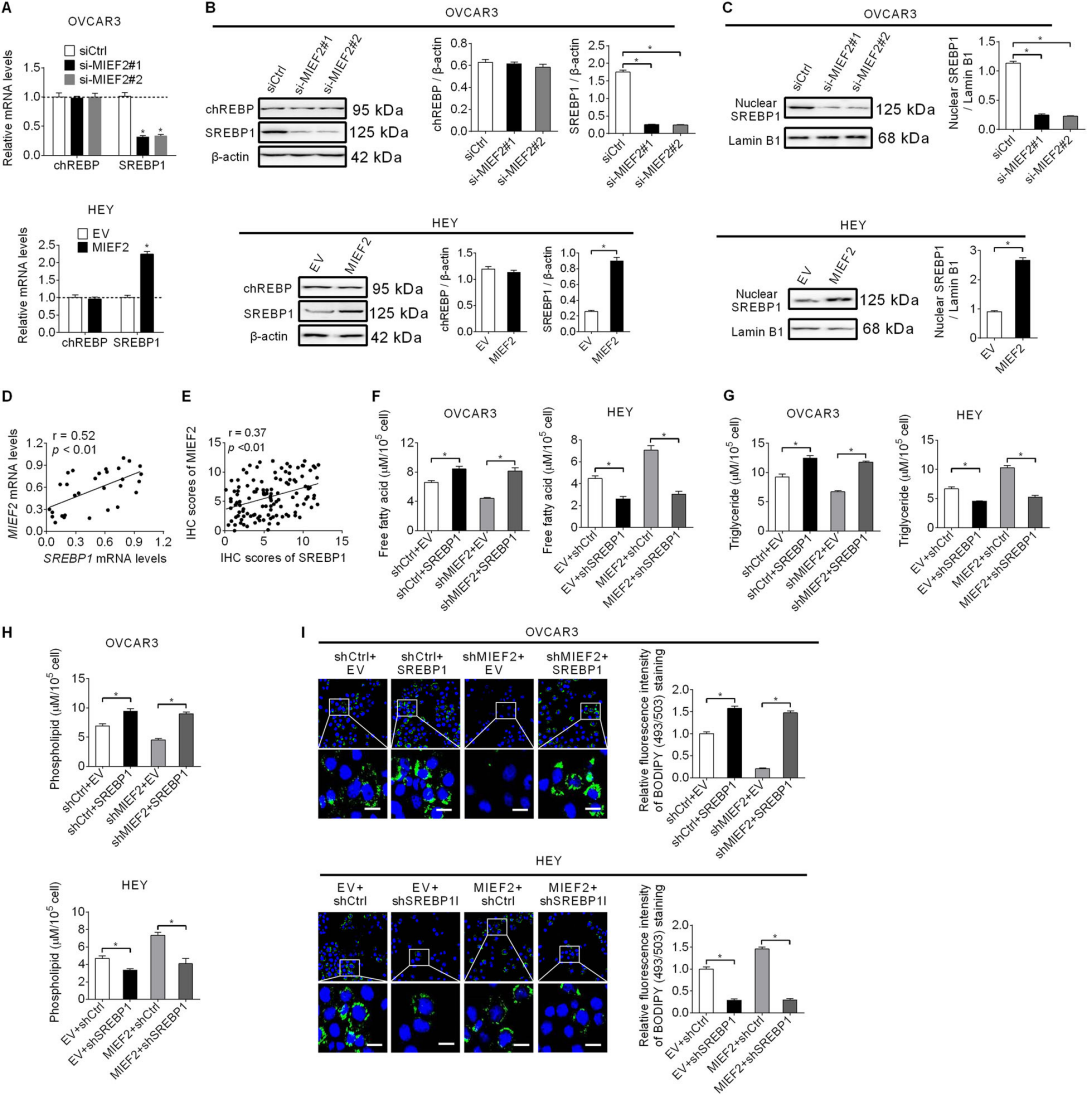
MIEF2 increased the expression levels of fatty acid synthesis enzymes via up-regulating SREBP1. **a**, **b** qRT-PCR and Western blot analysis for expressions of chREBP and SREBP1 in OVCAR3 and HEY cells with MIEF2 knocked-down or over-expressed (*N* = 3). **c** The nuclear expression level of SREBP1 was determined by western blot analysis in OVCAR3 and HEY cells with MIEF2 knocked-down or over-expressed (*N* = 3). **d** Spearman correlation analysis for the relationship between the mRNA expression levels of MIEF2 and SREBP1 in tumor tissues from 30 OC patients. **e** Spearman correlation analysis for the relationship between the protein expression levels of MIEF2 and SREBP1 in tumor tissues from another 122 OC patients. **f**–**h** Cellular content of free fatty acid (**f**), triglyceride (**g**) and phospholipids (**h**) were measured in OVCAR3 and HEY cells with MIEF2 knocked-down or over-expressed (*N* = 3). **i** Detection of neutral lipid content by fluorescence dye BODIPY 493/503 staining in OVCAR3 and HEY cells with MIEF2 knocked-down or over-expressed. Scale bars, 20 μm. The average fluorescence intensity per cell was analyzed using image J (*N* = 20 cells per group).

MIEF2 increased the expression levels of fatty acid synthesis enzymes (ACC1, FASN, SCD1) at both mRNA and protein levels, suggesting a regulation at transcriptional level. To explore the molecular mechanism by which MIEF2 up-regulated fatty acid synthesis enzymes (ACC1, FASN, SCD1), the levels of carbohydrate-responsive element-binding protein (chREBP) and sterol regulatory elementary binding protein 1 (SREBP1), crucial transcriptional regulators in the regulation of lipogenic gene expression^28^, were determined by qRT-PCR and Western blot analyses in OVCAR3 and HEY cells. No significant changes of chREBP were observed when MIEF2 was knocked-down or over-expressed, while SREBP1 was significantly decreased when MIEF2 was knocked-down, but increased when MIEF2 was over-expressed (Fig. 3a, b). As expected, the nuclear expression level of SREBP1, which represents its transcriptional activity, exhibited a similar expression pattern to that of total SREBP1, implying that MIEF2 activated the transcriptional activity of SREBP1 in OC cells (Fig. 3c). Spearman rank correlation analysis also revealed a positive correlation between the expression levels of MIEF2 and SREBP1 at both mRNA level by qRT-PCR analysis from 30 OC patients (*r* = 0.52, *p* < 0.01) (Fig. 3d) and protein level by immunohistochemistry staining assay (Supplementary Fig. S3) from another 122 OC patients (*r* = 0.37, *p* < 0.01) (Fig. 3e). We next investigate whether SREBP1 was involved in MIEF2-upregulated expressions of ACC1, FASN and SCD1 and fatty acid synthesis in OC cells. Our results revealed that MIEF2 knockdown significantly decreased the content of intracellular free fatty acid, triglyceride and phospholipids, as well as the intensity of BODIPY staining in OVCAR3 cells, whereas forced expression of SREBP1 markedly restored those reductions mediated by MIEF2 knockdown. By contrast, MIEF2 over-expression markedly increased the content of intracellular free fatty acid, triglyceride and phospholipids, as well as the intensity of BODIPY staining, while SREBP1 silencing remarkably attenuated the fatty acid synthesis-promoting effect of MIEF2 over-expression (Fig. 3f–i).

### MIEF2 increased the expression levels of cholesterol biosynthesis via up-regulating SREBP2

**Fig. 4:**
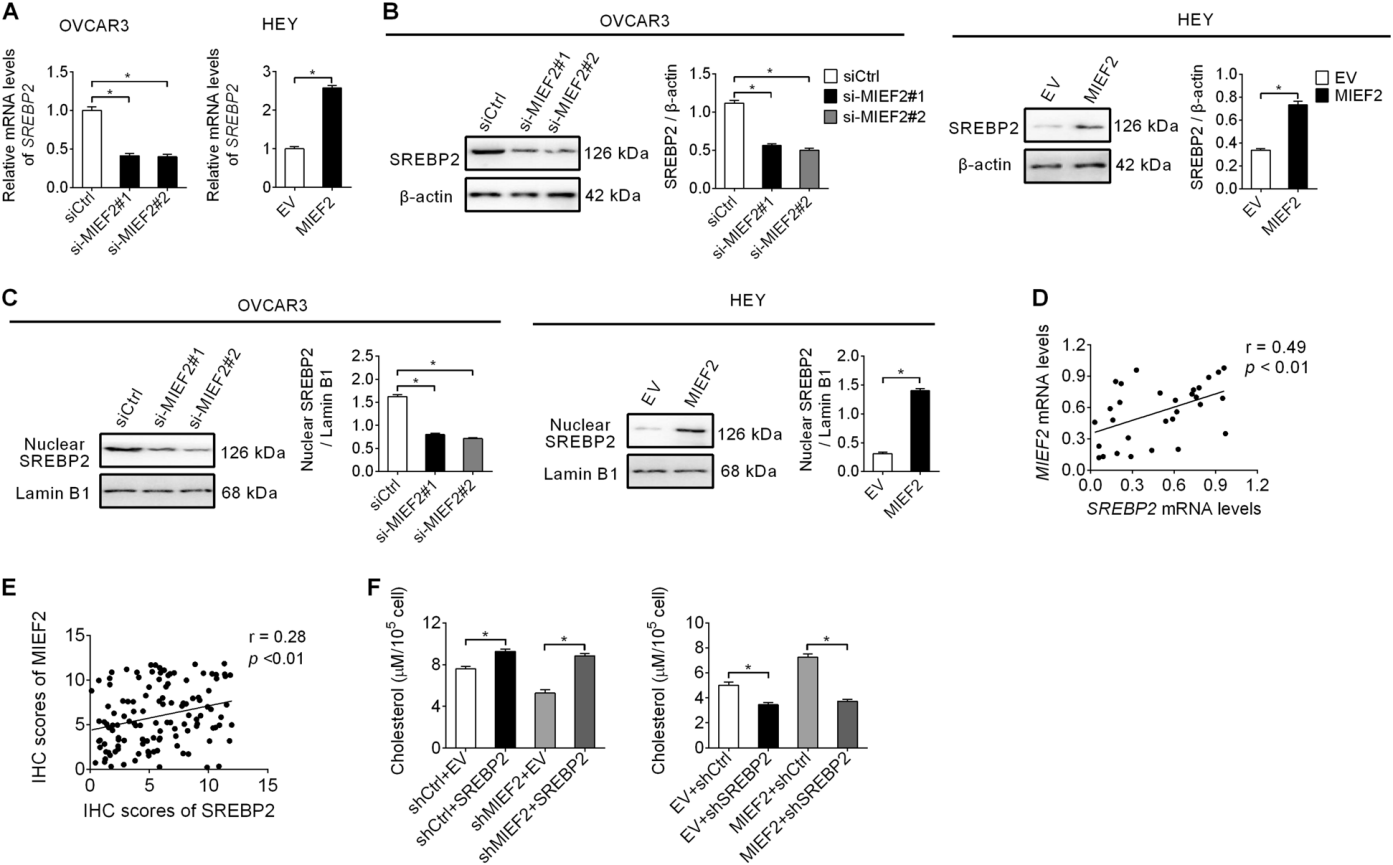
MIEF2 increased the expression levels of cholesterol biosynthesis via up-regulating SREBP2. **a**, **b** qRT-PCR and Western blot analysis for expression of SREBP2 in OVCAR3 and HEY cells with MIEF2 knocked-down or over-expressed (*N* = 3). **c** The nuclear expression level of SREBP2 was determined by western blot analysis in OVCAR3 and HEY cells with MIEF2 knocked-down or over-expressed (*N* = 3). **d** Spearman correlation analysis for the relationship between the mRNA expression levels of MIEF2 and SREBP2 in tumor tissues from 30 OC patients. **e** Spearman correlation analysis of the relationship between the protein expression levels of MIEF2 and SREBP2 in tumor tissues from another 122 OC patients. **f** Cellular content of cholesterol was measured in OVCAR3 and HEY cells with treatment as indicated (*N* = 3).

Next, we sought to determine the molecular mechanism by which MIEF2 up-regulated cholesterol biosynthesis enzymes HMGCS1 and HMGCR. Given that SREBP2 has been well established as a key transcriptional regulator in the regulation of cholesterol biosynthesis gene expression^29^, the effect of MIEF2 knockdown or over-expression on the expression level of SREBP2 was determined by qRT-PCR and Western blot analyses. Similar to the effect of MIEF2 on SREBP1, knockdown of MIEF2 also significantly suppressed SREBP2 expression at both mRNA and protein levels in OVCAR3 cells, while MIEF2 over-expression markedly increased SREBP2 expression in HEY cells (Fig. 4a, b). Additionally, the nuclear expression level of SREBP2, which represents its transcriptional activity, exhibited a similar pattern to the total SREBP2 expression (Fig. 4c), suggesting that MIEF2 activated the transcriptional activity of SREBP2 in OC cells. Furthermore, spearman rank correlation analysis revealed a positive correlation between the expression levels of MIEF2 and SREBP2 at both mRNA level by qRT-PCR analysis from 30 OC patients (*r* = 0.49, *p* < 0.01) (Fig. 4d) and protein level by immunohistochemistry staining assay (Supplementary Fig. S3) from another 122 OC patients (*r* = 0.28, *p* < 0.01) (Fig. 4e). We then explored whether SREBP2 was involved in MIEF2-mediated up-regulation of HMGCS1 and HMGCR, and cholesterol synthesis in OC cells. Our results showed that MIEF2 knockdown significantly reduced the intracellular levels of cholesterol, which were significantly restored by SREBP2 over-expression. Inversely, the intracellular cholesterol level were clearly increased when MIEF2 was over-expressed in HEY cells, which were remarkably attenuated by SREBP2 silencing (Fig. 4f).

### MIEF2 increased SREBP1 and SREBP2 by activating ROS/AKT/mTOR signaling

**Fig. 5:**
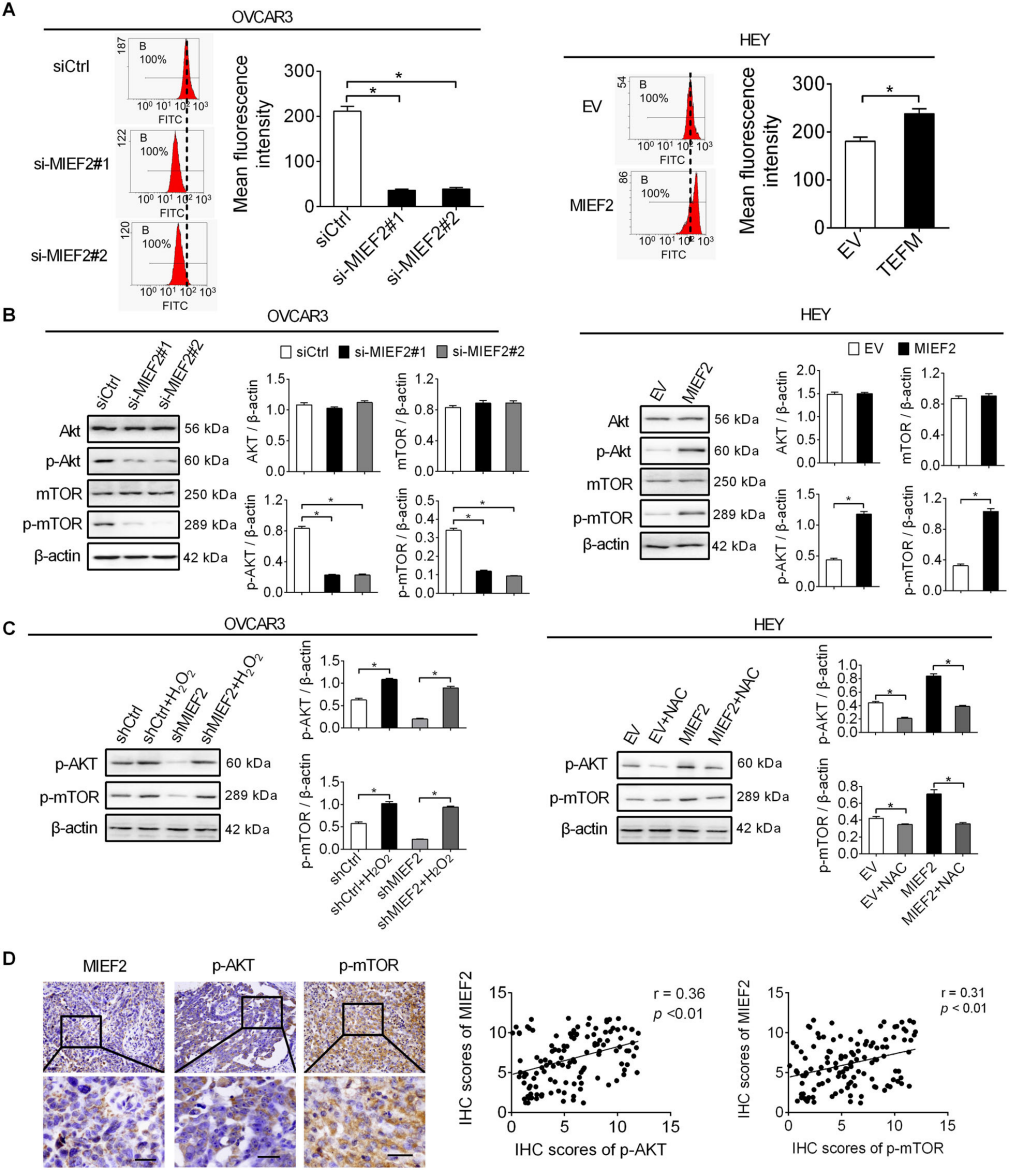
MIEF2 activated ROS/AKT/mTOR signaling in OC cells. **a** Intracellular ROS level was determined by flow cytometry in OVCAR3 and HEY cells with MIEF2 knocked-down or over-expressed (*N* = 3). **b** Western blot analysis for AKT, p-AKT, mTOR and p-mTOR in OVCAR3 and HEY cells with MIEF2 knocked-down or over-expressed (*N* = 3). **c** Western blot analysis for p-AKT and p-mTOR in OVCAR3 cells treated with 90 μM H_2_O_2_ for 12 h as indicated (*N* = 3). **d** Left panel: representative immunohistochemical (IHC) staining of MIEF2, p-AKT and p-mTOR in tumor tissues of OC. Scale bars, 50 μm. Right panel: Spearman correlation analysis of the relationship between the protein expression levels of MIEF2 and p-AKT and p-mTOR in tumor tissues from 122 OC patients.

Mitochondria are major source of reactive oxygen species (ROS), which activates multiple oncogenic signaling pathways, such as Akt signaling, during tumor progression^30^. Considering that Akt/mTOR pathway has been well established to play a central role in the regulation of cell lipid metabolism^31,32^, we hypothesized that MIEF2 overexpression may increase ROS production and thus activate Akt/mTOR pathway to promote the SREBP1- and SREBP2-mediated de novo fatty acid synthesis and cholesterol biosynthesis. MitoTracker Green staining assay showed that knockdown of MIEF2 lead to a dramatic mitochondrial elongation in OVCAR3 cells, while MIEF2 overexpression induced fragmentation of mitochondrial in HEY cells (Supplementary Fig. S4), as expected. Flow cytometry analysis revealed that knockdown of MIEF2 significantly reduced ROS levels in OVCAR3 cells, while forced MIEF2 expression significantly elevated ROS levels in HEY cells (Fig. 5a). Additionally, MIEF2 knockdown significantly decreased the phosphorylation levels of Akt (Ser473) and mTOR (Ser2448) in OVCAR3 cells, whereas MIEF2 over-expression exhibited the opposite effects on the phosphorylation levels of Akt (Ser473) and mTOR (Ser2448) in HEY cells (Fig. 5b), indicating that MIEF2 activates Akt/mTOR signaling in OC cells. To further test whether increased ROS level contributed to the activation Akt/mTOR signaling, H_2_O_2_ or NAC (an ROS scavenger) was added to change cellular ROS levels in OC cells. Our results showed that regulation of Akt/mTOR signaling by MIEF2 was markedly attenuated when OC cells were treated with H_2_O_2_ or NAC (Fig. 5c). Furthermore, spearman rank correlation analysis revealed a positive correlation between the expression levels of MIEF2 and phosphorylation levels of AKT and mTOR by immunohistochemistry staining assay from 122 OC patients (*r* = 0.36, *p* < 0.01; *r* = 0.31, *p* < 0.01) (Fig. 5d). These results suggest that MIEF2 activated ROS/AKT/mTOR signaling in OC cells.

**Fig. 6:**
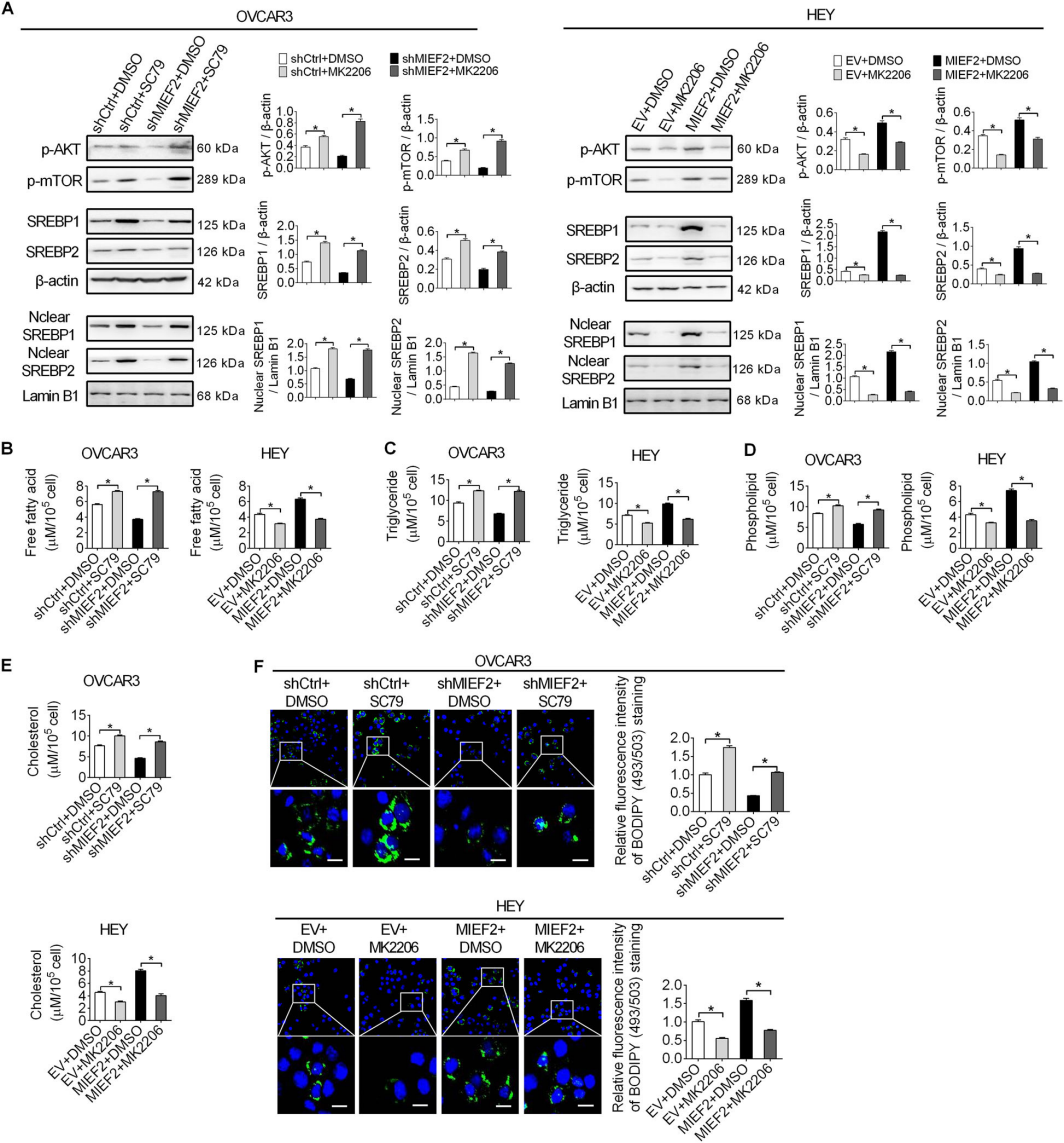
MIEF2 increased SREBP1 and SREBP2 by activating ROS/AKT/mTOR signaling. **a** Western blot analysis for total and phosphorylated Akt and mTOR, as well as total and nuclear expressions of SREBP1 and SREBP2 in OVCAR3 and HEY cells treated with MK2206 (5 µM for 24 h) or SC79 (10 µM for 24 h) (*N* = 3). **b**–**e** Cellular content of free fatty acid (**b**), triglyceride (**c**), phospholipids (**d**) and cholesterol (**e**) were detected in OVCAR3 and HEY cells treated with MK2206 (5 µM for 24 h) or SC79 (10 µM for 24 h) (*N* = 3). **f** Detection of neutral lipid content by fluorescence dye BODIPY 493/503 staining in OVCAR3 and HEY cells treated with MK2206 (5 µM for 24 h) or SC79 (10 µM for 24 h). Scale bars, 20 μm. The average fluorescence intensity (per cell) was analyzed with image J (*N* = 20 cells per group).

Next, to exam the involvement of AKT/mTOR signaling in MIEF2-promoted OC growth and metastasis, we treated OC cells with MK2206 (inhibitor of Akt) or SC79 (activator of Akt). Western blot analysis showed that MK2206 treatment markedly decreased the phosphorylation levels of Akt (Ser473) and mTOR (Ser2448), as well as both total and nuclear levels of SREBP1 and SREBP2 in HEY cells with or without MIEF2 overexpression. In contrast, SC79 treatment increased the phosphorylation levels of Akt (Ser473) and mTOR (Ser2448), as well as both total and nuclear levels of SREBP1 and SREBP2 in OVCAR3 cells with or without MIEF2 knockdown (Fig. 6a). Meanwhile, the levels of intracellular free fatty acid, triglyceride, phospholipids, cholesterol and BODIPY staining of neutral lipids were also decreased upon treatment with MK2206 in HEY cells, while increased upon SC79 treatment, as expected (Fig. 6b–f). Additionally, suppression of mTOR by treatment with rapamycin (specific inhibitor of mTOR) also markedly attenuated MIEF2 overexpression-increased lipid content in ovarian cancer cells, as evidenced by elevated intracellular levels of free fatty acid, triglyceride, phospholipids, cholesterol and enhanced BODIPY staining of neutral lipids in OC cells (Supplementary Fig. S5a–e). Together, these results indicate that MIEF2 increased SREBP1 and SREBP2 and thus fatty acid synthesis by activating ROS/AKT/mTOR signaling.

### MIEF2 promoted OC growth and metastasis by enhancing fatty acid and cholesterol biosynthesis

**Fig. 7:**
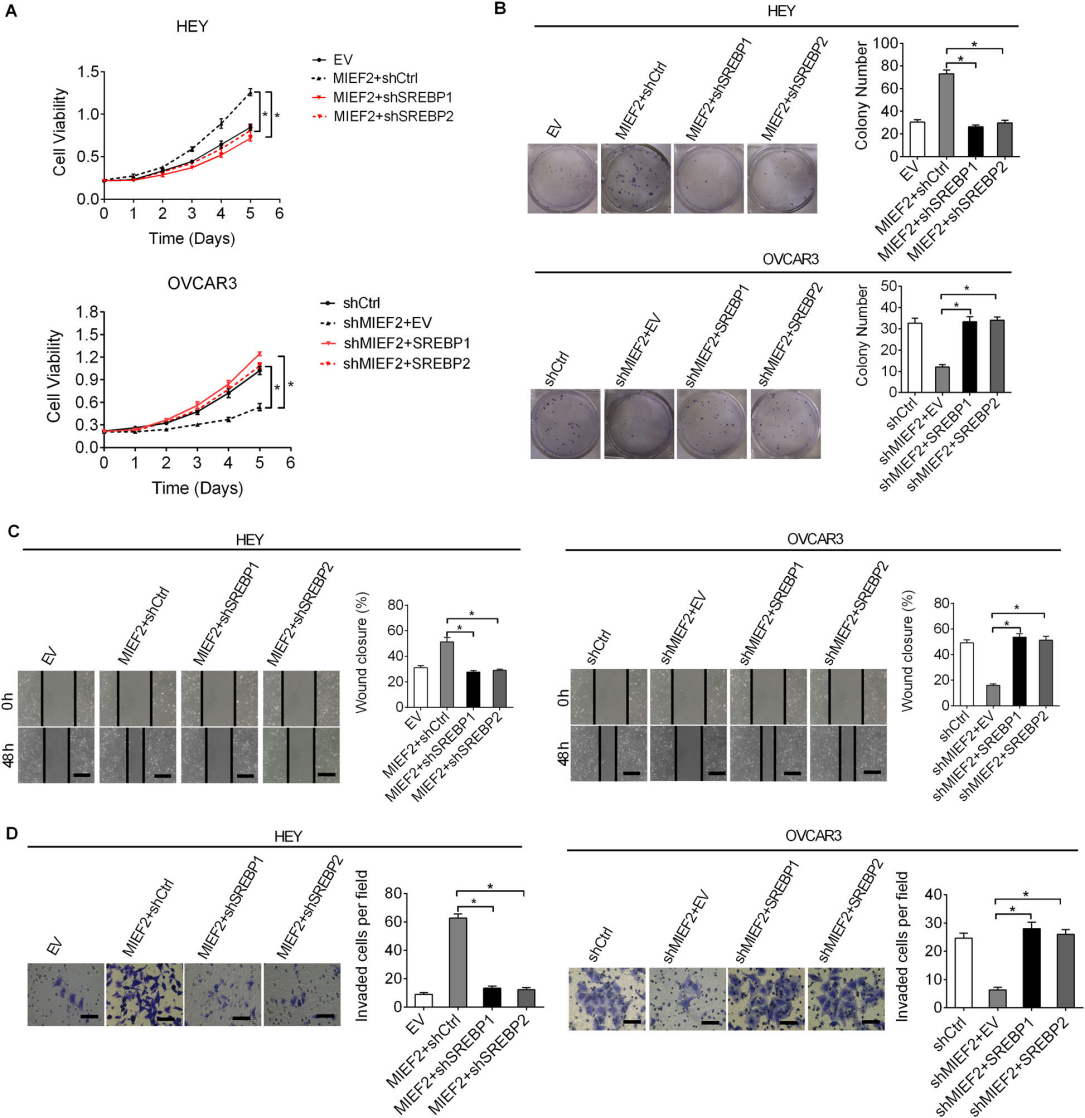
MIEF2 increased the expression levels of cholesterol biosynthesis via up-regulating SREBP2. **a** Cell proliferation was determined by the MTS assay in HEY and OVCAR3 cells treated as indicated (EV, empty vector; MIEF2, expression vector encoding MIEF2; shCtrl, control shRNA; shSREBP1, shRNAs against SREBP1; shSREBP2, shRNAs against SREBP2; SREBP1, expression vector encoding SREBP1; SREBP2, expression vector encoding SREBP2) (*N* = 3). **b** Colony formation assay in HEY and OVCAR3 cells with different treatment as indicated (*N* = 3). **c** Scratch wound healing assay for cell migration ability in HEY and OVCAR3 cells with different treatment as indicated (*N* = 3) Scale bars, 200 μm. **d** Transwell matrigel invasion assay for invasion ability in HEY and OVCAR3 cells with different treatment as indicated (*N* = 3) Scale bars, 50 μm.

Considering that increased fatty acid and cholesterol biosynthesis has been coupled with various malignant phenotypes of cancer cells, including tumor growth and metastasis, we therefore tested whether MIEF2 promote OC growth and metastasis through enhancing fatty acid and cholesterol synthesis. Our results showed that MIEF2 over-expression significantly promoted the proliferation, colony formation, migration and invasion abilities of OVCAR3 cells, which were attenuated by either knockdown of SREBP1 or SREBP2. In contrast, MIEF2 knockdown significantly suppressed the proliferation, colony formation, migration and invasion abilities of HEY cells (Fig. 7a–d), which were reversed by overexpression of either SREBP1 or SREBP2, suggesting that MIEF2 exerts its oncogenic role in OC cells by enhancing fatty acid and cholesterol biosynthesis.

## Discussion

Metabolic reprogramming has been established as a hallmark of cancer. Among metabolic alterations, increased *de novo* lipogenesis has been recognized as one of the important but not well-characterized hallmarks of cancer cells^33,34^. Previous studies mainly focused on the intricate relationship between oncogenic signaling and lipid metabolism reprogramming in cancer^35^, while as the crucial organelles involved in cellular metabolism regulation, the contributions of mitochondrial dysfunction to the reprogrammed lipid metabolism in cancer cells is still largely unknown, especially in ovarian cancer (OC). In the present study, we demonstrate that, as one of the key regulators of mitochondrial fission, MIEF2 significantly enhanced the fatty acid a synthesis and cholesterol biosynthesis in OC cells, which contributed to both tumor growth and metastasis. Mechanistically, we found that MIEF2 enhanced the lipid biosynthesis through up-regulating the expression of SREBP1 and SREBP2 and their transcriptional target lipogenic enzymes of ACC1, FASN, SCD1, HMGCS1 and HMGCR through increasing mitochondrial ROS production and subsequently activation of AKT/mTOR signaling pathway. Our results suggest that MIEF2 overexpression-mediated mitochondrial dysfunction plays a critical role in the reprogramming of lipid metabolism in ovarian cancer cells.

Increased *de novo* fatty acid (FA) synthesis has been observed in many different types of cancer and is currently thought to be the major metabolic pathway exploited by cancer cells for FA acquisition^36^. To date, increased expressions of key regulators of lipogenesis, including SREBPs, acetyl-CoA carboxylase (ACC), fatty acid synthase (FASN), and stearoyl-CoA desaturase 1 (SCD1) have been observed in various human cancers^1,9,10,15,35^. Previous studies in OC also have demonstrated the up-regulation of FASN and its involvement in tumor growth and metastasis^37^. In OC stem cells, stearoyl-CoA desaturease 1 (SCD1) regulated unsaturated fatty acids synthesis were shown to be essential for cell proliferation and survival, while inhibition of SCD1 eliminated ovarian cancer stem cells and retarded tumor initiation^38^. Consistently, our data show that MIEF2 promoted the *de novo* fatty acid synthesis of OC cells through up-regulation of the lipogenic enzymes of ACC1, FASN and SCD1, which further support the oncogenic role for dysregulated lipogenic enzymes in the promotion of cancer progression. Comparing with de novo fatty acid synthesis, cholesterol biosynthesis in cancer has received less attention. Elevated intracellular level of cholesterol has been demonstrated in several types of cancer^4,39^. The oncogenic roles of HMGCR, a rate-limiting enzyme in cholesterol biosynthesis, have been revealed in gastric cancer^40^, glioblastoma^41^ and prostate^42^ cancer cells. Inhibition of the enzymatic activity of HMGCR could result in increased DNA damage and reduced cell proliferation, adhesion and invasion in primary cell cultures of ovarian cancer^43^. Consistent with these results, our results also indicated that HMGCR played a crucial role in MIEF2-promoted cholesterol biosynthesis and thus the progression of OC. Compared with lipid biogenesis in tumorigenesis, the relevance of fatty acid oxidation (FAO) to cancer has received less attention. During the last few years, except for the lipid biogenesis in tumorigenesis, the importance of FAO in cancer metabolism is being increasingly recognized^44^. Many recent studies have shown that cancer cells rely on FAO for cell proliferation, metastasis, survival and drug resistance^6^. However, no significant effect of MIEF2 on fatty acid oxidation was observed in OC cells in our present study, suggesting a lipogenetic- but not lipolytic-promoting role played by MIEF2 in OC.

Sterol regulatory element-binding proteins (SREBPs) are central transcription factors that control the expression of lipid synthesis genes^8^. Three SREBP isoforms, including SREBP-1a, SREBP-1c and SREBP-2, have been identified in mammalian cells^15^. SREBP1 is relatively specific to the regulation of fatty acid synthesis, while SREBP2 is relatively specific to the regulation of cholesterol biosynthesis^7^. Previous studies have demonstrated increased expressions of SREBP1 and SREBP2 in several types of cancer, including OC^15,45^. Our findings showing that MIEF2 up-regulated SREBP1 and SREBP2 expressions in OC cells, further supporting the crucial roles played by both SREBP1-mediated de novo lipogenesis and SREBP2-mediated cholesterol biosynthesis in the progression of cancer. In addition, these results also suggest that simultaneous activations of de novo lipogenesis and cholesterol biosynthesis may be a frequently metabolic event in the progression of cancer, which still needs further investigation.

Mitochondrial malfunction is a critical step in the pathogenesis of many diseases such as neurodegenerative disease and cancer^46^. Mitochondrial dysfunction induced reactive oxygen species (ROS) production contributes to genomic instability and activation of multiple oncogenic signaling pathways^47,48^. Several previous studies have revealed that increased mitochondria fission could elevate the production of intracellular ROS in several types of cancers, including OC^49^. Similarly, we found that MIEF2 overexpression also promoted ROS production in OC cells, suggesting hyper-activation of mitochondrial fission is a major cause of mitochondrial ROS production in tumor cells. The AKT/mTOR pathway, a major downstream signaling activated by ROS production, has been shown to be frequently activated in several types of human cancers and prompted lipid synthesis through activating SREBP1/2^50^. Our present study indicated that the elevation of ROS production and subsequently activation of Akt/mTOR pathway was involved in the up-regulation of SREBP1 and SREBP2 by MIEF2. Recently, we have demonstrated that MIEF2 is frequently up-regulated and contributes to both growth and metastasis of ovarian cancer (OC) cells. Given that increased fatty acid and cholesterol biosynthesis provides not only building blocks but also signaling molecules that are required for cancer growth and metastasis, we therefore tested whether MIEF2-promoted lipid synthesis was involved in the growth and metastasis of OC. Expectedly, we found that either inhibition of SREBP1 or SREBP2 could robustly attenuated the growth and metastasis promoted by MIEF2 overexpression.

In conclusion, we demonstrate a crucial role for MIEF2 in the promotion of de novo fatty acid synthesis and cholesterol biosynthesis in OC cells, which provides novel insights to understand the underlying mechanisms of reprogrammed lipid metabolism in cancer cells, as well as a strong line of evidence for this molecule to be used as a drug target in the treatment of OC.

## Materials and methods

### Reagents

The siRNAs were purchased from Gene Pharma (China). MK2206 (#S1078), SC79 (#S786) and rapamycin (S1039) were purchased from Selleck Chemicals (Houston, TX, USA).

### Cell culture and tissue collection

Human ovarian cancer cell lines of HEY, SKOV3, ES2 and OVCAR3 were obtained from the Cell Bank of Chinese Academy of Sciences (Shanghai, China) and cultured in DMEM or RPMI-1640 medium containing 10% FBS. All cell lines were recently authenticated by STR DNA profiling and tested for mycoplasma contamination. Besides, 152 tumor tissue samples were collected from the Department of Gynaecology and Obstetrics at Xijing Hospital (30 for qRT-PCR analysis; 122 for IHC staining analysis). This study has been approved by the Ethics Committee of Xijing Hospital. Written informed consents were obtained from all individuals.

### Over-expression and knockdown of target genes

Silencing experiments were performed with the transfection of two different specific siRNAs (20 nM) against MIEF2 or a negative control siRNA using Lipofectamine 2000 (Invitrogen, California, USA) according to the manufacturer’s protocol. For construction of siRNA expression vectors, small hairpin RNA (shRNA) targeting SREBP1 (shSREBP1) and SREBP2 (shSREBP) was cloned into a pSilencer™ 3.1-H1 puro vector (Ambion, Austin, TX, USA. The sequences of siRNAs used in this study were listed in the Supplementary Table 1. For forced expression of MIEF2, SREBP1 and SREBP2, the open reading frame sequence of MIEF2 (Entrez Gene ID: 125170, 6720 and 6721) was amplified and cloned into a pcDNA^TM^3.1(C) vector (Invitrogen, V790-20).

### Quantitative real-time PCR (qRT-PCR)

Total RNA was extracted from OC cells with different treatment using the RNeasy minikit (QIAGEN). Then, reverse transcription was performed uisng 0.5-1 μg extracted RNA with a QuantiTect reverse transcription kit (QIAGEN) following the manufacturer’s instructions. PCR reactions were performed using SYBR Green mix (Takara). Relative mRNA expression levels of target genes were normalized to β-actin and calculated using the 2^−^^△△Ct^ method. The primer sequences used in this study were listed in the Supplementary Table 1.

### Nuclear, mitochondrial and cytoplasmic protein extraction

To detect nuclear translocations of SREBP1 and SREBP2, NE-PER nuclear and cytoplasmic extraction reagent (Thermo Scientific Pierce, UK) was used as per manufacturer’s instructions. The purified nuclear and cytoplasmic proteins were precipitated by acetone. Then, protein concentration was determined by bicinchoninic acid (BCA) assay followed by detection with western blot analysis.

To detect mitochondria and cytoplasmic MIEF2 expression, mitochondrial and cytoplasm were isolated from OC cells with a Mitochondria Isolation Kit (Beyotime, China) as per manufacturer’s instructions and then lyzed with RIPA lysis buffer. The protein concentration was determined by BCA assay followed by detection with western blot analysis.

### Western blot

Whole-cell or nucleus/cytosol fraction protein lysates from OC cells were subjected to 10% polyacrylamide SDS-PAGE gels and transferred onto PVDF membrane (Millipore). The membranes were blocked with 5% nonfat milk and probed with the appropriate primary antibodies (listed in the Supplementary Table 2) overnight at 4 °C. After washed three times in PBS containing 0.1% Tween 20, the membranes were probed with anti-mouse or anti-rabbit immunoglobulin coupled to horseradish peroxidase 2 h at 28 °C. The signaling was visualized with an ECL detection kit (Amersham Biosciences).

### Mitochondrial morphology

Mitochondrial morphology was assessed by Mito Tracker Green FM (Invitrogen, USA) staining according to the manufacturers’ protocols. Firstly, ovarian cancer cells with different treatment were seeded into confocal dishes. After incubating with Mito Tracker green in DMEM medium, cells were washed with PBS three times and counterstained with DAPI. Images were acquired with an Olympus FV-1000 confocal microscope and the length of mitochondrial was quantified using Image J.

### Quantification of free fatty acid, triglyceride, phospholipids and cholesterol

OC cells with different treatment were lysed in RIPA buffer for 40 min. Then, cell homogenates were prepared for lipids extraction using chloroform/methanol (2:1). The levels of free fatty acid, triglyceride, phospholipids and cholesterol were determined with EnzyChromTM free fatty acid, triglyceride, phospholipid assay and cholesterol kits (Bioassay Systems, Hayward, CA, USA) according to the manufacturers’ protocols, respectively.

### Quantification of neutral lipid

Cellular neutral lipids were stained with fluorescence dye BODIPY 493/503 (Invitrogen) according to the manufacturer’s instruction. Briefly, OC cells were seeded into 10-cm dish and fixed with 4% paraformaldehyde for 15 min. After that, OC cells were stained with BODIPY 493/503 (1 μg/mL) for 1 h at 37 °C. Images were acquired with an Olympus FV-1000 confocal microscope and the staining intensities of neutral lipids were quantified using Image J.

### Immunohistochemistry (IHC) analysis

Tissue sections were deparaffinized by baking slides at 65°C for 30 min and then rehydrated in series of ethanol solutions. Endogenous peroxidase activities were quenched by 3% hydrogen peroxide for 10 min. Antigen retrieval was performed by boiling slides for 20 min in citrate buffer. After that, slides were blocked in 4% BSA for 1 h and incubated with primary antibodies (Supplementary Table 2) overnight at 4 °C. The results were determined with an IHC detection kit (MXB, Fuzhou, China) following the manufacturer’s protocol. The staining intensity scores were independently evaluated by two observers.

### Determination of fatty acid oxidation and uptake

For determination of fatty acid oxidation, OC cells were seeded into 6-well plate and cultured overnight. Cells were then washed with HBSS and cultured in 1 ml HBSS containing 1 μCi [9, 10(n)−3H] oleic acid (Amersham Pharmacia Biotech, Italy) for 24 h. After that, chloroform/methanol (2:1) was used for extraction of the aqueous phase containing ^3^H_2_O. Radioactivity in each cell group was detected in 8 ml scintillation solution with a L6500 scintillation counter (Beckman Coulter, Brea, CA).

For determination of fatty acid uptake, OC cells were prepared as described above. One ml HBSS containing 1 μCi [9, 10(n)−3H] oleic acid was then added to cells and cultured for 24 h. After washing 3 times with HBSS, cells were lysed in 0.2 ml 5% SDS. Radioactivity in each cell group was detected in 8 ml scintillation solution with a L6500 scintillation counter (Beckman Coulter, Brea, CA).

### MTS cell viability and colony formation assay

For MTS cell viability assay, 1 × 10^3^ OC cells were plated in 96-well plates (020096, Xinyou Biotech, Hangzhou, China). Cell viability was measured at 0, 24, 48, 72 and 96 h after cell seeding using the CellTiter 96 Aqueous One Solution cell proliferation kit (Promega, G3581) according to the manufacturer’s instructions.

For colony formation assay, 100 OC cells were seeded into 6-well plates and cultured for about two weeks. Colonies were fixed in 4% paraformaldehyde for 15 min and stained with with 0.5% crystal violet. The number of colonies formed in each plate was manually counted using Image J.

### Wound-healing cell migration assay

OC cells were seeded into 6-well plates and cultured overnight. After grown to 90% confluence, scratching in the bottom of wells was applied with a plastic pipette tip. Images were captured with a light Olympus microscope at 0 and 48 hours after scratching. Relative migration distance of OC cells in each group was determined using Image J.

### Matrigel invasion assay

Matrigel invasion assay was performed using 24-well invasion chambers purchased from BD Biosciences. Briefly, 3 × 10^4^ OC cells were seeded to each insert in medium without FBS overnight. Medium containing 20% FBS was added to the bottom of the inserts. After incubation for 48 h, cells remaining above the insert membrane were removed by a sterile cotton swab. Cells invaded through the Matrigel to the bottom of the insert were fixed in 4% paraformaldehyde for 15 min and stained with 0.5% crystal violet. Penetrated cells were counted under a light Olympus microscope.

### Statistical analysis

Data were presented as the mean ± SEM. The SPSS software (17.0 version) was used for statistical analysis and *p* < 0.05 was considered as statistically significant (*). The student’s t-test was used to calculate p value between two groups, while one-way ANOVA with Tukey’s post-hoc test was used for the data which have more than two groups.

## Supplementary information

revised supplementary figures and tables
