# Supplementary material for: MIEF2 reprograms lipid metabolism to drive progression of ovarian cancer through ROS/AKT/mTOR signaling pathway
Source: Cell Death Dis. 2021 Jan 5;12(1):18. doi: 10.1038/s41419-020-03336-6 (PMC7791105; doi:10.1038/s41419-020-03336-6)
Supplement: Supplementary file 1 — revised supplementary figures and tables [file 41419_2020_3336_MOESM1_ESM.docx]

**Supplemental information**

**MIEF2 reprograms lipid metabolism to drive progression of ovarian cancer through ROS/AKT/mTOR signaling pathway**

**Supplemental figures**

**
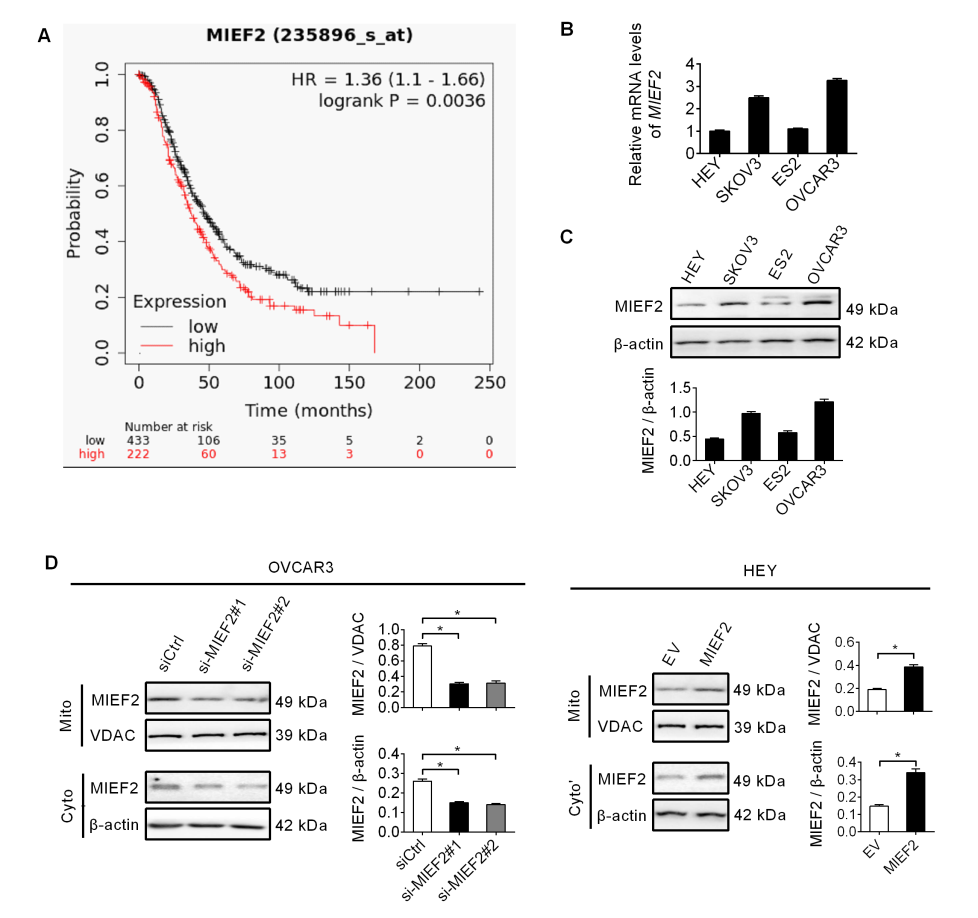
**

**Figure S1.** **(A)** Kaplan-Meier plotter^1^ analysis for clinical implication of MIEF2 in OC. **(B)** MIEF2 expression was determined by qRT-PCR (N=3)**.** (**C**) MIEF2 expression was determined by Western blot in four ovarian cancer cell lines. (**D**) MIEF2 expression in cytoplasmic (cyto) and mitochondrial (mito) fractions of OC was determined by Western blot in OVCAR3 and HEY cells when MIFF2 was knocked-down or over-expressed.

**
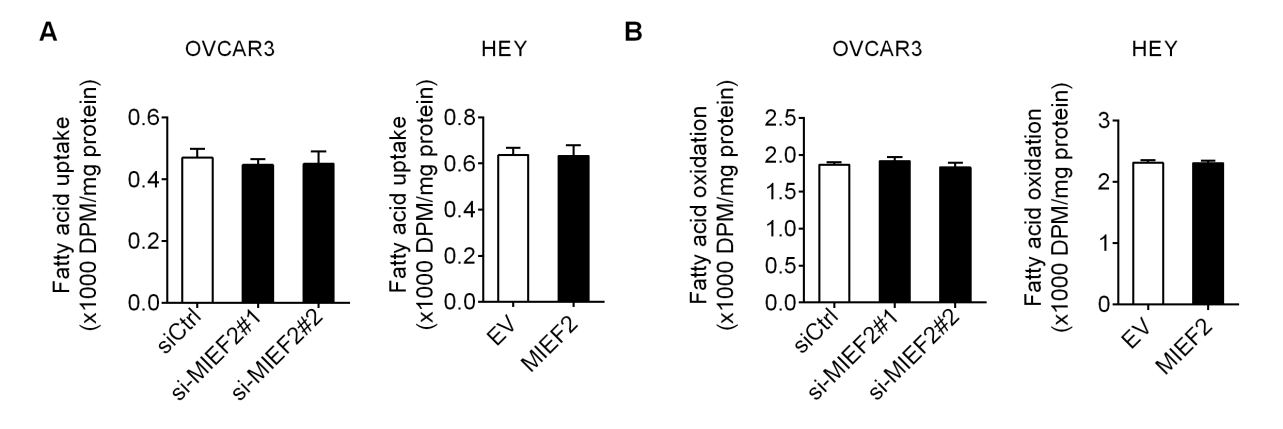
**

**Figure S2.** ^3^H-labeled oleic acid was used to determine the rate of fatty acid uptake **(A)** and oxidation **(B)** in OVCAR3 and HEY cells with MIEF2 knocked-down or over-expressed (N=3).

**
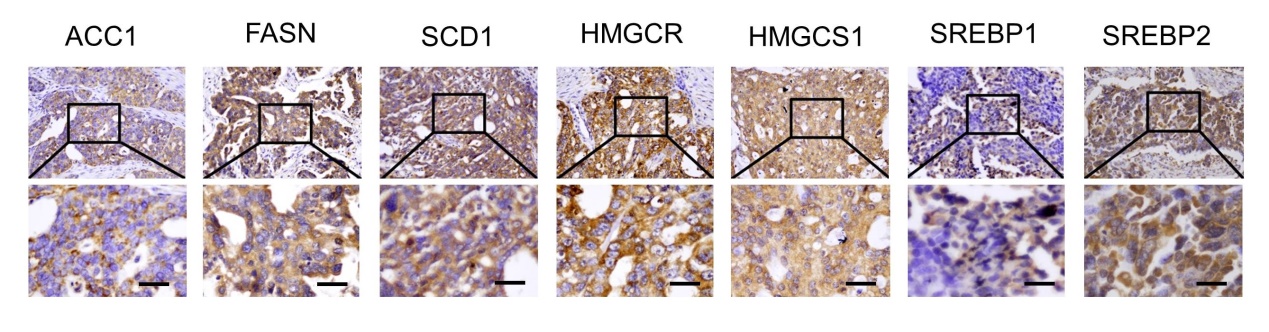
**

**Figure S3.** Representative immunohistochemical (IHC) staining images of ACC1, FASN, SCD1, HMGCR, HMGCS1, SREBP1 and SREBP2 in tumor tissues of OC.

**
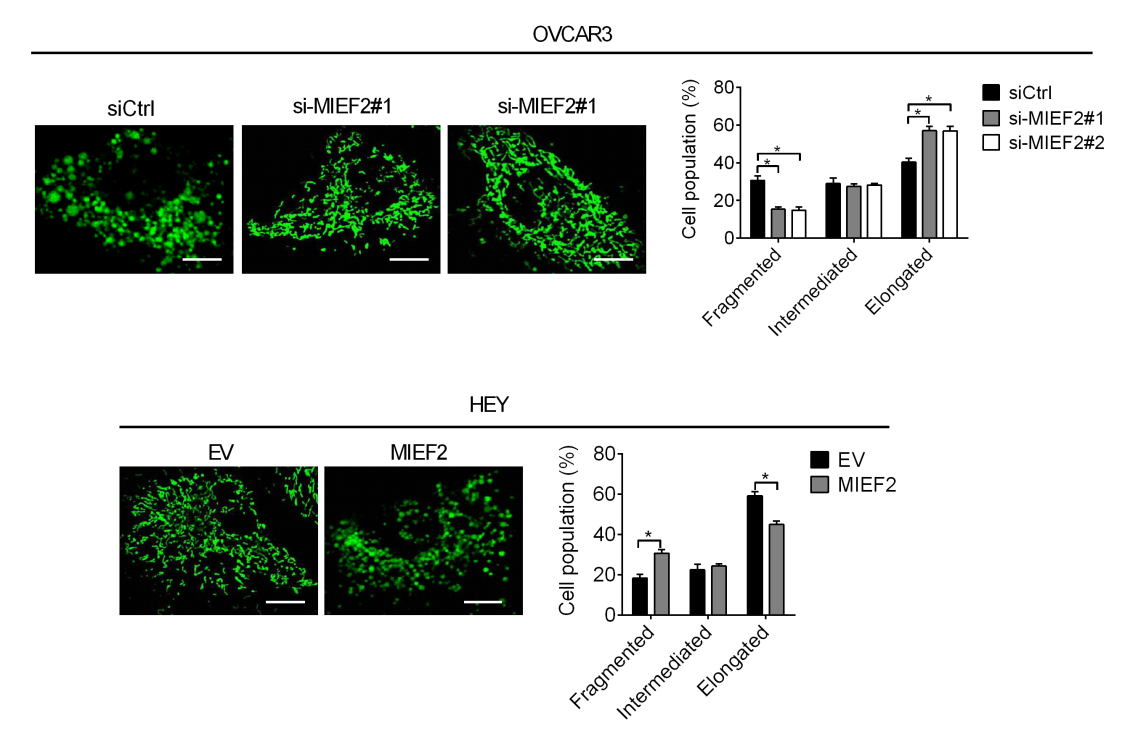
**

**Figure S4.** Representative mitochondrial morphology images of OVCAR3 and HEY cells with MIEF2 knocked-down or over-expressed. Scale bars, 5 μm. (N= 50 cells per group) Cells with elongated, intermediate and fragmented mitochondria were quantified as previously described^2^.

**
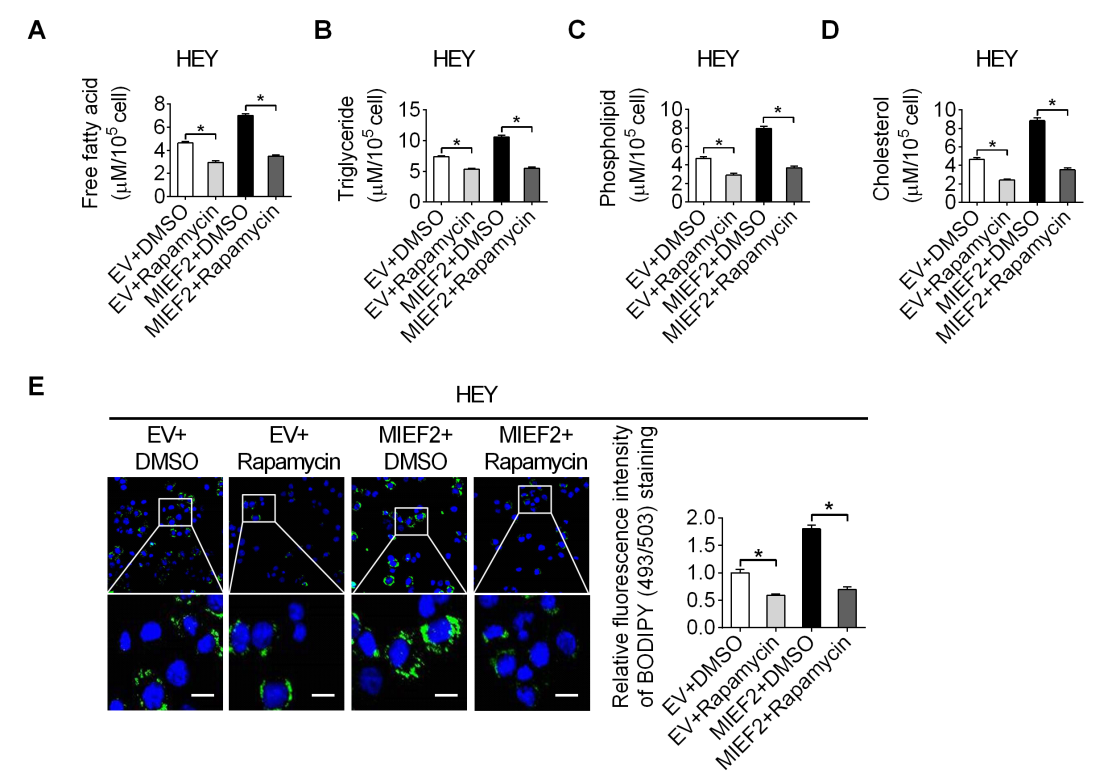
**

**Figure S5. Activation of mTOR is involved in MIEF2 overexpression-increased lipid content in ovarian cancer cells. (A-D)** Cellular content of free fatty acid **(A)**, triglyceride **(B)**, phospholipids **(C)** and cholesterol **(D** were detected in HEY cells treated with Rapamycin (0.1 µM for 24 h) (N=3). **(E)** Detection of neutral lipid content by fluorescence dye BODIPY 493/503 staining in HEY cells treated with Rapamycin (5 µM for 24 h). Scale bars, 20 μm. The average fluorescence intensity (per cell) was analyzed with image J (N=20 cells per group).

**Supplementary Tables**

**Table 1.** Sequence of primers for qRT-PCR analysis and siRNAs for knockdown of genes expression.

| **1. Primers used in q-PCR analysis** | | |
| --- | --- | --- |
| **Gene** | **Forward Primer** | **Reverse Primer** |
| *MIEF2* | CAGAAACGGGGGAAGCGG | CACCAGGAGACGCACATGG |
| *ACACA* | ATGTCTGGCTTGCACCTAGTA | CCCCAAAGCGAGTAACAAATTCT |
| *FASN* | AAGGACCTGTCTAGGTTTGATGC | TGGCTTCATAGGTGACTTCCA |
| *SCD1* | AGAATGGAGGAGATAAGT | TAGCAGAGACATAAGGAT |
| *HMGCS1* | GCTCTGGAATCTGGAATG | CTCTTCAATGGCAGTGTT |
| *HMGCR* | AAGAAGACAGCCTGAATAG | ATCCTCCACAAGACAATG |
| CD36 | GGCTGTGACCGGAACTGTG | AGGTCTCCAACTGGCATTAGAA |
| *CPT1A* | ATCAATCGGACTCTGGAAACGG | TCAGGGAGTAGCGCATGGT |
| *ACOX1* | TGGCGGACATGGCTATTCTCAC | TGGCTGGGCAGGTCATTCAAG |
| *SREBP1* | ACAGTGACTTCCCTGGCCTAT | GCATGGACGGGTACATCTTCAA |
| *SREBP2* | AACGGTCATTCACCCAGGTC | GGCTGAAGAATAGGAGTTGCC |
| *β-actin* | AGGCACCAGGGCGTGAT | GCCCACATAGGAATCCTTCTGAC |
| **2. siRNAs** | | |
| **Target** | **Forward** | **Reverse** |
| *MIEF2#1* | GCGCUAUACAGUGGCCUACAGGAGC | GCUCCUGUAGGCCACUGUAUAGCGCAU |
| *MIEF2#2* | AAAAACCACUAUUCAAAUCCC | GAUUUGAAUAGUGGUUUUUCU |
| *SREBP1* | CGGAGAAGCUGCCUAUCAATT | UUGAUAGGCAGCUUCUCCGTT |
| *SREBP2* | GCGCUCUCAUUUUACCAAATT | GGAUGAUGCCAAAGGUCAAATT |
| siCtrl | UUCUCCGAACGUGUCACGUTT | ACGUGACACGUUCGGAGAATT |

**Table 2.** Primary antibodies used in this study.

| **Antibody** | **Company (Cat. No.)** | **Working dilutions** |
| --- | --- | --- |
| MIEF2 | abcam (ab101350); abcam (ab247033) | WB: 1/1000; IHC:1/200 |
| VDAC | Proteintech (10866-1-AP) | WB: 1/1000 |
| ACC1 | Proteintech (21923-1-AP) | WB: 1/500; IHC:1/300 |
| FASN | Proteintech (10624-2-AP) | WB: 1/500; IHC:1/200 |
| SCD1 | abcam (ab23331) | WB: 1/1000; IHC:1/200 |
| HMGCS1 | abcam (ab155787) | WB: 1/1000; IHC:1/100 |
| HMGCR | abcam (ab242315) | WB: 1/1000; IHC:1/200 |
| CD36 | abcam (ab133625) | WB: 1/1000 |
| CPT1A | Proteintech (15184-1-AP) | WB: 1/1000 |
| ACOX1 | Proteintech (10957-1-AP) | WB: 1/1000 |
| SREBP1 | Proteintech (14088-1-AP); Proteintech (28212-1-AP) | WB: 1/1000; ; IHC:1/200 |
| SREBP2 | abcam (ab28482) | WB: 1/1000; ; IHC:1/300 |
| Lamin B1 | abcam (ab16048) | WB: 1/1000 |
| p- Akt  (Ser473) | Cell signaling (#4060S) | WB: 1/1000; IHC:1/200 |
| p-mTOR  (Ser2448) | abcam (ab109268) | WB: 1/1000; IHC:1/100 |
| β-actin | Proteintech (20536-1-AP) | WB: 1/1000 |

**Reference**

1 Nagy, A., Lanczky, A., Menyhart, O. & Gyorffy, B. Validation of miRNA prognostic power in hepatocellular carcinoma using expression data of independent datasets. *Scientific reports* **8**, 9227, doi:10.1038/s41598-018-27521-y (2018).

2 Gao, T. *et al.* SIK2 promotes reprogramming of glucose metabolism through PI3K/AKT/HIF-1alpha pathway and Drp1-mediated mitochondrial fission in ovarian cancer. *Cancer letters* **469**, 89-101, doi:10.1016/j.canlet.2019.10.029 (2020).
